# Clinical Characteristics and Prognostic Factors of Endometrial Cancer Patients With Liver Metastasis: A Surveillance, Epidemiology, and End Results Database (SEER)-Based Study of 1,034 Women

**DOI:** 10.7759/cureus.54606

**Published:** 2024-02-21

**Authors:** Abdulhameed Alhadeethi, Ahmed A Ibrahim, Ahmed Atia, Yasmeen J Alabdallat, Ibraheem M Alkhawaldeh, Mostafa H El Din Moawad

**Affiliations:** 1 Department of General Medicine, Al-Salam Teaching Hospital, Mosul, IRQ; 2 Cardiology, Faculty of Medicine, Menofia University, Menofia, EGY; 3 Cardiovascular Disease, Kasr Alainy School of Medicine, Cairo University, Cairo, EGY; 4 Surgery, Hashemite University, Zarqa, JOR; 5 Faculty of Medicine, Mutah University, Alkarak, JOR; 6 Clinical Epidemiology and Clinical Trials Management, Public Health and Community Department, Suez Canal University, Ismailia, EGY

**Keywords:** tumor, overall survival, seer, endometrial cancer, liver metastasis

## Abstract

Background

There are several patterns of metastatic spread from endometrial cancer (EC). Although studies have been conducted to study the EC population with distant metastasis in the bone and lungs, there is still a lack of studies on liver metastasis. This study aims to evaluate and assess the clinical features and prognostic factors of EC patients with liver metastasis.

Methodology

We conducted a retrospective cohort study adhering to the guidelines for reporting observational research. We utilized the Surveillance, Epidemiology, and End Results database to gather data on female patients diagnosed with EC and reported liver metastasis. We estimated survival curves using the Kaplan-Meier method and evaluated differences in survival using the log-rank test. We also conducted univariable and multivariable Cox proportional hazards regression analyses to determine the hazard ratios with 95% confidence intervals for overall survival (OS) and identify factors that impact survival.

Results

We analyzed data from 1,034 EC patients with liver metastasis. Median OS after liver metastasis was six months, and cancer-specific survival was seven months. Univariate Cox regression analysis revealed several factors associated with decreased OS in EC patients. These included age (≥60 years), non-endometrioid and sarcoma histological subtypes, absence of surgery, no chemotherapy, and the presence of distant metastasis to the lung, brain, and bone. Conversely, married marital status and white race were linked to a better prognosis. Subsequent multivariate Cox regression analysis identified age (≥60 years), non-endometrioid histological subtype, absence of surgery, no chemotherapy, and the presence of distant metastasis to lung, brain, and bone remaining as independent risk factors for decreased OS. In contrast, the white race still emerged as an independent prognostic factor for better OS.

Conclusions

Various risk factors, such as age, race, lung, bone, or brain metastasis, as well as chemotherapy and surgery, may influence the prognosis of individuals with primary EC liver metastases.

## Introduction

Endometrial cancer (EC) is the most common cancer affecting the female reproductive system in developed countries [1]. Of all cancer cases in women in the United States, EC is considered to be the fourth most common cancer in terms of incidence and the sixth most common cancer in terms of mortality [2]. EC is associated with many risk factors such as old age, white race, obesity, hormone replacement therapy, nullipara, and genetic predisposition [2,3].

Most EC cases are detected early, and International Federation of Gynecology and Obstetrics (FIGO) stages I or II are usually associated with a good prognosis, with a five-year survival rate of approximately 80% [4,5]. However, if detected late, patients usually present with distant metastasis (FIGO stage IVC), which is associated with a poor prognosis. Nearly 5-10% of EC cases are diagnosed as stage IVB, with a five-year survival rate of less than 10% despite receiving proper treatment [4,5].

Although distant organ metastasis in EC is uncommon, it has significant repercussions and is linked to significantly reduced lifespan. It is crucial to comprehend the patterns of metastasis in newly diagnosed cases to more correctly forecast prognosis and choose an effective care strategy. In addition, the precise site of distant metastasis can affect the survival of women with EC [6].

In a recent study of 2,948 women with EC, distant metastasis was identified in 1,796 patients, with liver metastasis emerging as the second most common site after lung metastasis. Specifically, among these cases of distant metastasis, 177 patients presented with single-site liver metastasis [6]. Several studies have been conducted to evaluate the different histopathological, clinical status, and treatment outcomes of the EC population. Studies have also been conducted to study the EC population with distant metastasis in the bone and lungs; however, there is still a lack of studies on liver metastasis [7]. This study was performed using the Surveillance, Epidemiology, and End Results (SEER) database to evaluate and assess the clinical features and prognostic factors of EC patients with liver metastasis.

## Materials and methods

Study design

We conducted a retrospective cohort study adhering to the guidelines for reporting observational research, as specified in the Strengthening the Reporting of Observational Studies in Epidemiology statement [8].

Data source and eligibility criteria

We utilized the SEER database (seer.cancer.gov) to gather data on female patients diagnosed with EC and reported liver metastasis. To access this information, we used SEER*Stat software version 8.3.5, which allowed us to explore the SEER 17 registries (2019 submission). The SEER database, established by the National Cancer Institute, is a publicly accessible and nationally representative resource that covers about 26.5% of the U.S. population across various geographic areas.

Our study focused on female patients diagnosed with EC and known liver metastasis between 2010 and 2016. The inclusion criteria required that patients have a histologically confirmed diagnosis. We excluded patients with missing information regarding marital status, tumor grade, survival duration, surgical intervention status, or radiation treatment.

Study variables

We gathered information on various factors such as age at diagnosis (divided into two age groups: below 60 years and above 60 years), marital status, race, and year of diagnosis. Additionally, we collected data on tumor characteristics, including tumor grade, histological type, surgery, chemotherapy, radiation, brain metastases, liver metastases, lung metastases, survival months, and vital status. The histological grade was classified into the following four grades: Grade I, Grade II, Grade III, and Grade IV. These grades are based on the level of differentiation observed in the tumor cells, with Grade I being well-differentiated and Grade IV being undifferentiated. Additionally, tumor histologic subtypes were categorized in accordance with International Classification of Diseases for Oncology codes as endometrioid subtypes (8380-8383), non-endometrioid subtypes (8000, 8010, 8013, 8020, 8022, 8033, 8041, 8045, 8046, 8050, 8070-8072, 8130, 8140, 8210, 8246, 8255, 8260, 8263, 8310, 8323, 8441, 8460-8461, 8480, 8482, 8560, 8570, and 8574-8575), and sarcomas (8800 and 8890-8999).

Outcome of interest

The primary objective of this analysis was overall survival (OS), which was defined as the time of diagnosis to the date of death or the date of the patient’s last contact (December 31, 2016). Patients were considered to have been censored if they were alive until the date of their last contact. Deaths that occurred within 30 days of diagnosis were recorded as 0 in the SEER database and it was converted to 0.5 in accordance with standard epidemiologic methods [9]. Causes of death are reported in the SEER database according to the International Classification of Diseases 3.

Statistical analysis

For descriptive statistics, we used frequency and percentage for dichotomous variables. To examine the relationship between dichotomous variables, we used the chi-square test for individuals. We estimated survival curves using the Kaplan-Meier method and evaluated differences in survival using the log-rank test. We also conducted univariable and multivariable Cox proportional hazards regression analyses to determine the hazard ratios (HRs) with 95% confidence intervals (CIs) for OS and identify factors that impacted survival. We used jamovi version 2.3 and Stata/MP version 14.0 for all statistical analyses. We used two-tailed statistical tests and considered a p-value lower than 0.05 to indicate statistical significance.

## Results

Characteristics of study populations

According to our inclusion and exclusion criteria, 1,034 female patients with EC complicated with liver metastasis were identified out of 109,625 total patients with EC. Of these, 697 (67.4%) patients were aged 60 years or above. Most of our patients were white (699, 67.6%), and the majority were unmarried (624, 60.3%). Tumor grades were identified as well differentiated (32, 3.1%), moderately differentiated (68, 6.6%), poorly differentiated (313, 30.3%), which was the most prevalent tumor grade in this cohort, and undifferentiated (165, 16.0%). Regarding histological subtypes, most of our patients had a non-endometrioid subtype (723, 69.9%), while 290 (28.0%) had an endometrioid subtype. Surgery was performed in 473 (45.7%) patients, while 174 (16.8%) patients received radiotherapy. Regarding metastases, 427 (41.3%) were to the lungs, 41 (4.0%) to the brain, and 186 (18.0%) to the bones. Moreover, 51.7% (535 cases) presented with liver metastasis as a sole site. Notably, when examining cases with metastases involving multiple sites, liver metastasis featured prominently. The combination of liver and lung metastases occurred in 28.3% (293 cases), while liver metastasis coupled with brain involvement was observed in 0.6% (6 cases). Additionally, 5.8% (60 cases) exhibited concurrent liver and bone metastases. Furthermore, in instances of three-site involvement, liver metastasis in combination with lung and brain metastases occurred in 1.4% (14 cases), with liver, lung, and bone involvement in 10.1% (105 cases), and liver metastasis alongside brain and bone metastases in 0.6% (6 cases). The most extensive multi-site metastases, involving the liver, lung, brain, and bone, were found in 1.4% (15 cases). Table 1 shows the characteristics and clinicopathological features of the study population.

**Table 1:** Summary of baseline characteristics of the study patients. *: Refers to American Indian/Alaska Native, Asian, or Pacific Islander. The data are presented as counts (N) and percentages (%).

Variable	Overall (N = 1,034)
Age group (year)	
<60	337 (32.6%)
≥60	697 (67.4%)
Race	
Black	226 (21.9%)
White	699 (67.6%)
Others*	109 (10.5%)
Marital status	
Married	410 (39.7%)
Others	624 (60.3%)
Grade	
Well-differentiated	32 (3.1%)
Moderately differentiated	68 (6.6%)
Poorly differentiated	313 (30.3%)
Undifferentiated	165 (16.0%)
Unknown	456 (44.1%)
Histological subtype	
Endometrioid	290 (28.0%)
Non-endometrioid	723 (69.9%)
Sarcoma	21 (2.0%)
Surgery	
Yes	473 (45.7%)
No	561 (54.3%)
Radiation	
Yes	174 (16.8%)
No	860 (83.2%)
Chemotherapy	
Yes	619 (59.9%)
No	415 (40.1%)
Lung metastasis	
No	607 (58.7%)
Yes	427 (41.3%)
Brain metastasis	
No	993 (96.0%)
Yes	41 (4.0%)
Bone metastasis	
No	848 (82.0%)
Yes	186 (18.0%)

Survival analysis using Kaplan-Meier curves

**Figure 1:**
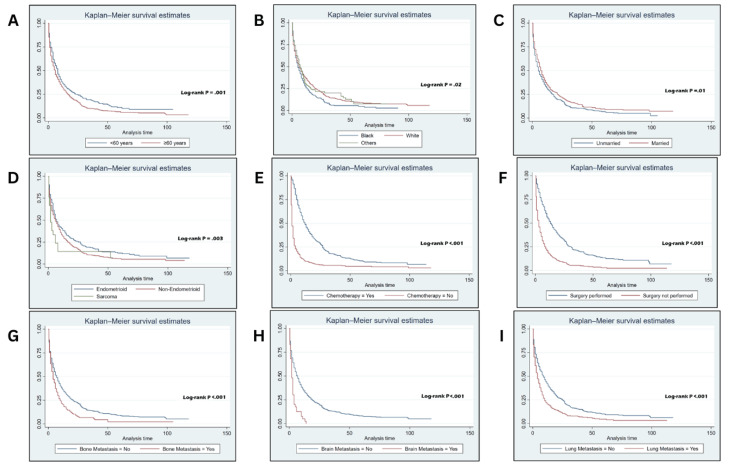
Endometrial cancer with liver metastasis overall survival using the Kaplan-Meier technique. Endometrial cancer with liver metastasis overall survival using the Kaplan-Meier technique, stratified by age group (A), race (B), marital status (C), histological subtypes (D), chemotherapy (E), surgery (F), bone metastasis (G), brain metastasis (H), and lung metastasis (I). Significance at p-values <0.05.

Median OS after liver metastasis in this cohort was six months, while cancer-specific survival (CSS) after liver metastasis was seven months. However, the slightly extended median CSS of seven months provides a nuanced perspective. CSS focuses specifically on survival attributed to the cancer itself, excluding deaths from other causes. This distinction suggests that despite the aggressive nature of liver metastasis in EC, patients may experience a period of disease-specific survival beyond the median OS. Age groups were identified to have a crucial role in determining OS among this cohort. Patients aged below 60 years were found to have a better prognosis than those aged 60 years or above with a median survival time of eight and six months, respectively. Kaplan-Meier curves shown in Figure 1 present the OS among this cohort and the effect of multiple variables on OS. Several prognostic factors were found to affect survival significantly, and some of them were associated with a significant decrease in OS such as age ≥60 years (log-rank p = 0.001), black race (log-rank p = 0.02), being unmarried (log-rank p = 0.001), having sarcoma histological subtype (log-rank p = 0.003), and metastasis to the lung, bone, or brain (log-rank p < 0.001). Moreover, patients who had not undergone surgical intervention or chemotherapy were found to be associated with a significant decrease in OS among this cohort (log-rank p < 0.001). Regarding radiotherapy, there was no statistically significant difference in OS between patients who received radiotherapy and those who did not (log-rank p > 0.05).

Prognostic and risk factors by Cox regression analysis

To determine the impact of various variables on OS, univariable regression was performed. Analysis showed that age ≥60 years (HR [95% CI] = 1.25 [1.086-1.442]; p = 0.002), non-endometrioid and sarcoma histological subtypes (HR [95% CI] = 1.23 [1.067-1.435]; p = 0.005 and HR [95% CI] = 1.70 [1.065-2.714]; p = 0.02, respectively), no surgery (HR [95% CI] = 2.35 [2.058-2.701]; p < 0.001), no chemotherapy (HR [95% CI] = 2.95 [2.574-3.382]; p < 0.001), and presence of distant metastasis to the lung (HR [95% CI] = 1.55 [1.362-1.777]; p < 0.001), brain (HR [95% CI] = 2.33 [1.691-3.217]; p < 0.001), and bone (HR [95% CI] = 1.44 [1.223-1.708]; p < 0.001) were important indicators of OS decline. By contrast, married marital status (HR [95% CI] = 0.85 [0.745-0.974]; p = 0.01) and white race (HR [95% CI] = 0.81 [0.698-0.957]; p = 0.01) were found to be associated with a better prognosis. These variables were subsequently analyzed using multivariate Cox regression to further identify the independent prognostic factors. Analysis revealed that age ≥60 years (HR [95% CI] = 1.20 [1.037-1.390]; p = 0.01), non-endometrioid histological subtype (HR [95% CI] = 1.22 [1.046-1.440]; p = 0.01), no surgery (HR [95% CI] = 2.02 [1.745-2.341]; p < 0.001), no chemotherapy (HR [95% CI] = 2.75 [2.384-3.179]; p < 0.001), presence of distant metastasis to the lung (HR [95% CI] = 1.24 [1.086-1.438]; p = 0.002), brain (HR [95% CI] = 1.79 [1.286-2.513]; p < 0.001), and bone (HR [95% CI] = 1.22 [1.026-1.466]; p = 0.02) were independent risk factors that decreased OS among this cohort. On the other hand, white race (HR [95% CI] = 0.83 [0.709-0.982]; p = 0.03) was found to be an independent prognostic factor for better OS. Further details about univariate and multivariate analysis are shown in Table 2.

**Table 2:** Univariate and multivariate Cox proportional hazards regression analyses of predictors of overall survival in the study patients. *: Refers to American Indian/Alaska Native, Asian, or Pacific Islander. Data are presented as hazard ratios (HRs) and 95% confidence intervals (CIs). Significance at p-value <0.05.

	Univariate analysis			Multivariate analysis		
	HR	P-value	95% CI	HR	P-value	95% CI
Age (year)						
<60	1			1		
≥60	1.25	0.002	1.086-1.442	1.20	0.014	1.037-1.390
Marital status						
Unmarried	1			1		
Married	0.85	0.01	0.745-0.974	0.93	0.34	0.816-1.073
Race						
Black	1			1		
White	0.81	0.01	0.698-0.957	0.83	0.03	0.709-0.982
Others*	0.80	0.08	0.629-1.028	0.94	0.66	0.736-1.216
Grade						
Well-differentiated	1			1		
Moderately differentiated	1.31	0.26	0.811-2.115	1.70	0.02	1.055-2.763
Poorly differentiated	1.55	0.04	1.015-2.378	2.01	0.002	1.302-3.113
Undifferentiated	1.37	0.15	0.886-2.136	1.84	0.008	1.173-2.912
Unknown	1.42	0.09	0.935-2.178	1.60	0.03	1.043-2.477
Histological type						
Endometrioid	1			1		
Non-endometrioid	1.23	0.005	1.067-1.435	1.22	0.01	1.046-1.440
Sarcoma	1.70	0.02	1.065-2.714	1.42	0.14	0.883-2.305
Surgery performed						
Yes	1			1		
No	2.35	0.001	2.058-2.701	2.02	0.001	1.745-2.341
Radiation						
Yes	1			1		
No	1.03	0.69	0.870-1.232	1.17	0.08	0.978-1.412
Chemotherapy						
Yes	1			1		
No or unknown	2.95	0.001	2.574-3.382	2.75	0.001	2.384-3.179
Lung metastasis						
No	1			1		
Yes	1.55	0.001	1.362-1.777	1.24	0.002	1.086-1.438
Brain metastasis						
No	1			1		
Yes	2.33	0.001	1.691-3.217	1.79	0.001	1.286-2.513
Bone metastasis						
No	1			1		
Yes	1.44	0.001	1.223-1.708	1.22	0.02	1.026-1.466

## Discussion

Although distant metastasis is uncommon in EC, liver metastasis is the second most prevalent organ metastatic site for EC with a five-year OS of 8% against 78% for patients who have or do not have liver metastases, respectively, according to earlier research [6,10]. To our knowledge, this population-based analysis is the first and largest to present prognostic factors related to primary EC liver metastasis, with the aim of enhancing assessment and therapy choices in these patients.

Previous studies reported poor survival for elderly women with EC [10-13]. In general, advanced-aged women with EC had poorer pathologic traits such as higher tumor grade and depth of myometrial invasion. Moreover, older women are more likely to be diagnosed with a late-stage disease. The results of our study also prove the significant association between the higher age group (age ≥60 years) as an independent risk factor and decreased OS among the study population that was consistent with a previous study which reported age to decrease OS in EC patients with distant metastasis [10].

Earlier research suggested that married women have longer OS, perhaps because partners could give social assistance and motivate patients to get medical care [10,14-16]. In our study, being unmarried exhibited significance in univariable Cox regression, but its significance diminished in the multivariable model. This suggests potential confounding or interaction effects with other covariates.

Our findings suggest that being white is an independent predictor that anticipates improved OS. The survival relative difference between black and white women could be due to more advanced stage, high-grade, and aggressive histologic subtype cancers, as well as unfairness in medical service delivery, including differential adoption of surgery in black women, as discussed by previous studies [17,18].

In the study cohort, those who did not get surgical intervention or chemotherapy had a lower OS rate. Radiotherapy, on the other hand, played no role in improving OS in these patients. A previous study found that chemotherapy was superior to radiotherapy following primary surgery in advanced stages of EC [19].

Other locations of distant organ metastasis, such as the lung, brain, or bone, were also significant predictors of lower OS. These findings are consistent with a prior study that found individuals with multiple metastatic sites of primary EC had worse survival outcomes [20].

The multivariate analyses in our study revealed that the non-endometrioid histological subtype emerged as an independent risk factor for lower OS within our study cohort. This finding aligns with the conclusions drawn by Liu et al. and Hu et al. [6,7], supporting the notion that the non-endometrioid subtype is characterized by more aggressive biological behavior and limited responsiveness to targeted medicines and hormonal therapy when compared to endometrioid EC. Furthermore, the increased susceptibility of non-endometrioid tumors to harbor mutations in the *TP53* gene is noted, which, in turn, can contribute to a poorer prognosis [21,22].

Our study contributes valuable information on the prognosis of EC patients with liver metastasis in a large number of patients. However, certain limitations of this study must be acknowledged. To begin, the retrospective design of this study, with all of its constraints, should not be overlooked. Second, because SEER data is collected from a modest number of sites within the United States, data cannot be generalized to other sites or countries. Third, SEER data lacks precise clinical information such as comorbidities, performance state, or treatment-related hazards, all of which can influence cancer prognosis. In addition, some variables such as the size of liver metastasis were not included in the analysis due to a high proportion of missing data, which likely compromised the reliability and interpretability of the results. Future studies with improved data completeness are encouraged. Fourth, SEER data is often collected for a short period, which may not capture long-term outcomes or late therapeutic effects.

We recommend further longitudinal studies to investigate the OS of EC patients with liver metastasis with adequate sample size and expanding the investigation of broader variables that may affect the OS rates.

## Conclusions

Various risk factors, such as age, race, tumor histological subtype, lung, bone, or brain metastasis, as well as chemotherapy and surgery, may influence the prognosis of individuals with primary EC liver metastases. These variables may be of significant assistance in practice to better evaluate women with this condition, as well as decide an appropriate treatment plan and prioritize patients based on the extent to which they benefit from the medical service.
